# *Dlg1* deletion in microglia ameliorates chronic restraint stress induced mice depression-like behavior

**DOI:** 10.3389/fphar.2023.1124845

**Published:** 2023-02-22

**Authors:** Xiaoheng Li, Zhixin Peng, Lingling Jiang, Ping Zhang, Pin Yang, Zengqiang Yuan, Jinbo Cheng

**Affiliations:** ^1^ The Brain Science Center, Beijing Institute of Basic Medical Sciences, Beijing, China; ^2^ Hengyang Medical College, University of South China, Hengyang, Hunan, China; ^3^ School of Basic Medical Sciences, Anhui Medical University, Hefei, Anhui, China; ^4^ Center on Translational Neuroscience, College of Life and Environmental Science, Minzu University of China, Beijing, China

**Keywords:** *Dlg1*, microglia, neuroinflammation, chronic restraint stress, depression

## Abstract

**Background:** Major depression is one of the most common psychiatric disorders worldwide, inflicting suffering, significant reduction in life span, and financial burdens on families and society. Mounting evidence implicates that exposure to chronic stress can induce the dysregulation of the immune system, and the activation of brain-resident innate immune cells, microglia, leading to depression-like symptoms. However, the specific mechanisms need to be further elucidated.

**Method:** Animal models of depression were established by chronic restraint stress (CRS), and depression-like behavior was assessed by sucrose preference test (SPT), open field test (OFT), tail suspension test (TST) and forced swimming test (FST). Microglial activation was visualized by immunofluorescent and immunohistochemical staining, and microglial morphological changes were further analyzed by skeleton analysis. The levels of inflammatory cytokines were detected by western blotting and qPCR.

**Result:** Microglial *Dlg1* knockout ameliorates CRS-induced mice depression-like behavior. In contrast to the effect of *Dlg1* in the LPS-induced mouse model, *Dlg1* knockout had little effect on microglial density, but significantly decreased the number of activated microglia and reversed microglia morphological changes in mice challenged with CRS. Moreover, the upregulation of inflammatory cytokines following CRS exposure was partially reversed by *Dlg1* deletion.

**Conclusion:** Our study provides the evidence that *Dlg1* ablation in microglia remarkedly reverses microglial activation and depression-like behavior in mice exposed to CRS, implicating a potential target for the treatment of clinical depression.

## Introduction

Major depression, a common psychiatric disorder throughout the world, is a leading cause of disability, with an estimated 5.0% of the adults suffering, according to the WHO. As a major contributor to suicide, especially in adolescent, major depression also increases susceptibility to other illnesses with much poorer prognosis (Benros et al., 2013). Despite the recent decades witnessed advances in the understanding of major depression, the underlining mechanism remains to be elucidated. Cumulative evidence shows that depression and neuroinflammation have reciprocal connections (Wohleb et al., 2016; Beurel et al., 2020). Inflammation is involved in depression, and depression can be induced by immune challenge, the way between depression and immune-activation is bidirectional (Licinio and Wong, 1999; Dowlati et al., 2010; Wong et al., 2016; Colasanto et al., 2020; Cao et al., 2021). Accordingly, studies aiming at neuroinflammation might provide valuable insights into the understanding of major depression.

Neuroinflammation, termed as pathological immune processes within the central nervous system (CNS), is mainly mediated by microglia. Evidence demonstrates that microglial activation can be induced by stress exposure, which has been recognized as a trigger for depression in human (McLaughlin et al., 2010; Park et al., 2015) or depression-like behavior in animals (Yang et al., 2015; Hao et al., 2019; Song et al., 2020). As the innate immune cells in the CNS parenchyma, microglia not only play vital roles in immune surveillance and defense, but also in brain development, homeostatic maintenance, as well as disease initiation and progression (Sierra et al., 2019; Wright-Jin and Gutmann, 2019; Lukens and Eyo, 2022). The delicate ramified processes and highly motile identity allow microglia to sense and react to the local environment (Umpierre and Wu, 2021). During stress exposure, microglia response to these neuronal changes as well as neurotransmitters and hormone alterations, subsequently contribute to stress-induced neuroinflammation, thus shaping neuronal function and associated behavior (Wohleb et al., 2016; Schramm and Waisman, 2022). However, the complexity of stress conditions and communication between microglia and other cells make the exact mechanism of microglial activation remains mostly unclear.

Discs large homolog 1 (Dlg1), a member of the membrane-associated guanylate kinase family, which has been reported to be involved in the peripheral immune response and to be a risk factor for neuropsychiatric disorders (Oliva et al., 2012; Zanin-Zhorov et al., 2012; Gupta et al., 2018). In our previous study, we demonstrated that microglial Dlg1 is a regulator of NF-κB signaling, and *Dlg1* deletion inhibits microglial activation and inflammatory cytokine production, thereby alleviating LPS-induced depression behavior (Peng et al., 2021). Despite being considered an acute model of depression, pain, lethargy, hypersensitivity, and reduced social interaction induced by LPS challenge are often considered sickness behaviors, which are hard to differentiate from depression-like behaviors (Dantzer et al., 2008; Wohleb et al., 2016). We therefore employed chronic restraint stress (CRS) model to induce depressive like behavior in mice in this study, and found that microglia activated upon CRS exposure, and *Dlg1* deletion in microglia significantly reverses this activation and thus ameliorates depressive like behavior induced by CRS. Collectively, our studies elucidate that *Dlg1* microglia-specific knockout not only mitigates inflammation-initiated depression, but also stress-induced depression as well as associated microglial activation.

## Materials and methods

### Mice

*CX3CR1* ^*CreER*^ mice were purchased from the Jackson Laboratory (Sacramento, CA, United States). *Dlg1* ^*flox/flox*^ mice were gifted by Dr. Wanli Liu (Tsinghua University, China) as previously described (Peng et al., 2021). To induce *Dlg1* microglia-specific knockout, tamoxifen (S1238, Selleck) was given to mice at the age of 6-week by gavage, and each mouse was administrated a total dose of 18 mg of tamoxifen for three consecutive days. CRS was performed 6 weeks after tamoxifen administration followed by behavioral and pathological analysis. All mice were housed in the Animal Care Facility at the Institute of Basic Medical Sciences with free access to standard rodent chow and clean water. All animal experiments were approved by the Institutional Animal Care and Use Committee of the Beijing Institute of Basic Medical Sciences.

### CRS procedures

Chronic restraint stress (CRS) was modified from the procedure previously described (Li et al., 2018). Briefly, the mice were placed in a 50 mL conical tube, with 0.5 cm air holes for breathing, for 3 h per day for 14 consecutive days.

### RNA isolation and qPCR

Total RNA from brain tissue was extracted using TRIzol™ reagent (# 15596018, Invitrogen). cDNA was synthesized with a One-step First-strand cDNA synthesis kit (AE311-03, TransGen Biotech, Beijing, China). qPCR was performed using 2 × SYBR green master mix to measure the expression of genes. The primer sequences for qPCR are as follows:

*GAPDH* forward: 5′-AGG​TCG​GTG​TGA​ACG​GAT​TTG-3′;

*GAPDH* reverse: 5′-TGT​AGA​CCA​TGT​AGT​TGA​GGT​CA-3′;

*TNFα* forward: 5′-CCC​TCA​CAC​TCA​GAT​CAT​CTT​CT-3′;

*TNFα* reverse: 5′-GCT​ACG​ACG​TGG​GCT​ACA​G-3′;

*Il-6* forward: 5′-CCA​AGA​GGT​GAG​TGC​CTT​CCC-3′;

*Il-6* reverse: 5′-CTG​TTG​TTC​AGA​CTC​TCT​CCC​T-3′;

*Il-1β* forward: 5′-GCA​ACT​GTT​CCT​GAA​CTC​AAC​T-3′;

*Il-1β* reverse: 5′-ATC​TTT​TGG​GGT​CCG​TCA​ACT-3′.

### Western blotting

The hippocampal tissue was lysed on ice using RIPA lysis buffer containing a cocktail of protease and phosphatase inhibitors. The lysates were centrifuged at ×15,000 g for 15 min at 4°C, then supernatants were collected and protein concentration was determined by BCA assays and was adjusted to the same final concentration. After heat denaturation, samples were separated by SDS-PAGE and transferred to Nitrocellulose membranes which were blocked in 5% non-fat milk and subsequently incubated overnight with the primary antibody. Relevant Horseradish Peroxidase (HRP)-conjugated secondary antibodies were incubated, and the immunoreactive proteins were then detected using an enhanced chemiluminescent (ECL) substrate. The protein signal was visualized using an X-Ray Film Processor (Optimax, New York, United States). The following antibodies were used: anti-iNOS/NOS Type II (# 610332, BD Biosciences, San Jose, CA, United States), anti- Iba1 (019-19741, Wako, Richmond, VA, United States), anti-Gapdh (#CW0266A, CWBiotech, Beijing, China).

### Immunohistochemistry and immunofluorescence

Mice were anesthetized, perfused and sacrificed. Brains were then removed, fixed in 4% paraformaldehyde for 48 h, and dehydrated with gradient sucrose in phosphate buffered saline (PBS). The brains were embedded in optimal cutting temperature compound (OCT) and sectioned on a freezing microtome (CM3050S, Leica, Wetzlar, Germany). The coronal sections (40 μm in thickness) were incubated overnight with anti-goat Iba1 antibody (1:400, 019-19741, Wako, Richmond, VA, United States) and anti-mouse Gfap antibody (1:400; MAB360, Millipore, Darmstadt, Germany).

For immunohistochemical staining, the slices were incubated with biotinylated immunoglobulin G (IgG), followed by streptavidin-conjugated horseradish peroxidase (VECTASTAIN ABC Kit, Zhongshan Jinqiao, Beijing, China), and finally was visualized by reacting with hydrogen peroxide (DAB Kit, Zhongshan Jinqiao). Images were captured using a Leica SCN400 scanner.

For immunofluorescent staining, the sections were labeled with TRITC AffiniPure goat anti-rabbit IgG (111-025-003, Jackson ImmunoResearch) fluorescent secondary antibody, and the images were acquired by Nikon confocal microscope (Nikon, Melville, NY, United Stats). For activated microglia quantification, we determined the activated microglia by larger soma area, shorter and bolder processes.

### Behavioral tests

For all behavioral tests, mice, in their home cages, were habituated for 2 h in the quiet and dimly lit test room before testing.

### Open field test (OFT)

During the test, spontaneous activity of mice was monitored for a 5-min period as they moved in the apparatus (50 × 50 × 20 cm), time spent in the central area and total distances traveled were analyzed by ANY-maze software.

### Tail suspension test (TST)

Mice were suspended by the tails on the instrument with a clip (50 cm above the floor) using a medical tape placed 1 cm from the tip of the tail. The test was recorded for 6 min and the immobility time was measured in the last 4 min of the test.

### Forced swimming test (FST)

Mice were allowed to swim in water for 2 min the day before the test for habituation. During the test, mice were placed in a clear glass cylinder (diameter 25 cm), which was filled with water (21°C–23°C) up to a height of 25 cm from the bottom. The test was recorded for 6 min and the immobility time was measured in the last 4 min of the test.

### Sucrose preference test (SPT)

The sucrose preference test is performed to evaluate anhedonia in mice. Briefly, mice were given two identical standard drinking bottles and habituated to two bottles filled with water (1 day) and with 1% sucrose water (1 day) for 2 days. After being deprived of food and water for 12 h, mice had free access to two drinking bottles, one containing 1% sucrose and the other with normal drinking water for 5 days. The positions of these two bottles were switched every 12 h to avoid side preferences. The overall sucrose and water intakes were measured, and the sucrose preference rate was calculated as 100% × (sucrose intake/sucrose intake + water intake).

### Microglial skeleton analysis

Skeleton analysis of microglia was performed as described in our previous study (Cheng et al., 2021). Briefly, microglial images were obtained on Nikon A1 Confocal Microscope with ×20 objective at ×5 zoom. Skeletonize [two-dimensional (2D)/3D] plugin and Analyze Skeleton plugin of the software ImageJ were used for the skeleton analysis.

### Statistical analysis

Data are expressed as mean ± SEM of at least two independent experiments. Statistical differences between the two groups were analyzed using Student’s t-test. Statistical differences for multiple comparisons were analyzed by application of ordinary two-way ANOVA (Tukey’s multiple comparison test). Statistical significance was determined as follows: *p < 0.05, **p < 0.01, and ***p < 0.001. GraphPad Prism software (version 9.0, GraphPad, San Diego, United States) was used for statistical analysis.

## Results

### *Dlg1* depletion in microglia alleviates CRS induced mice depression-like behavior

**FIGURE 1:**
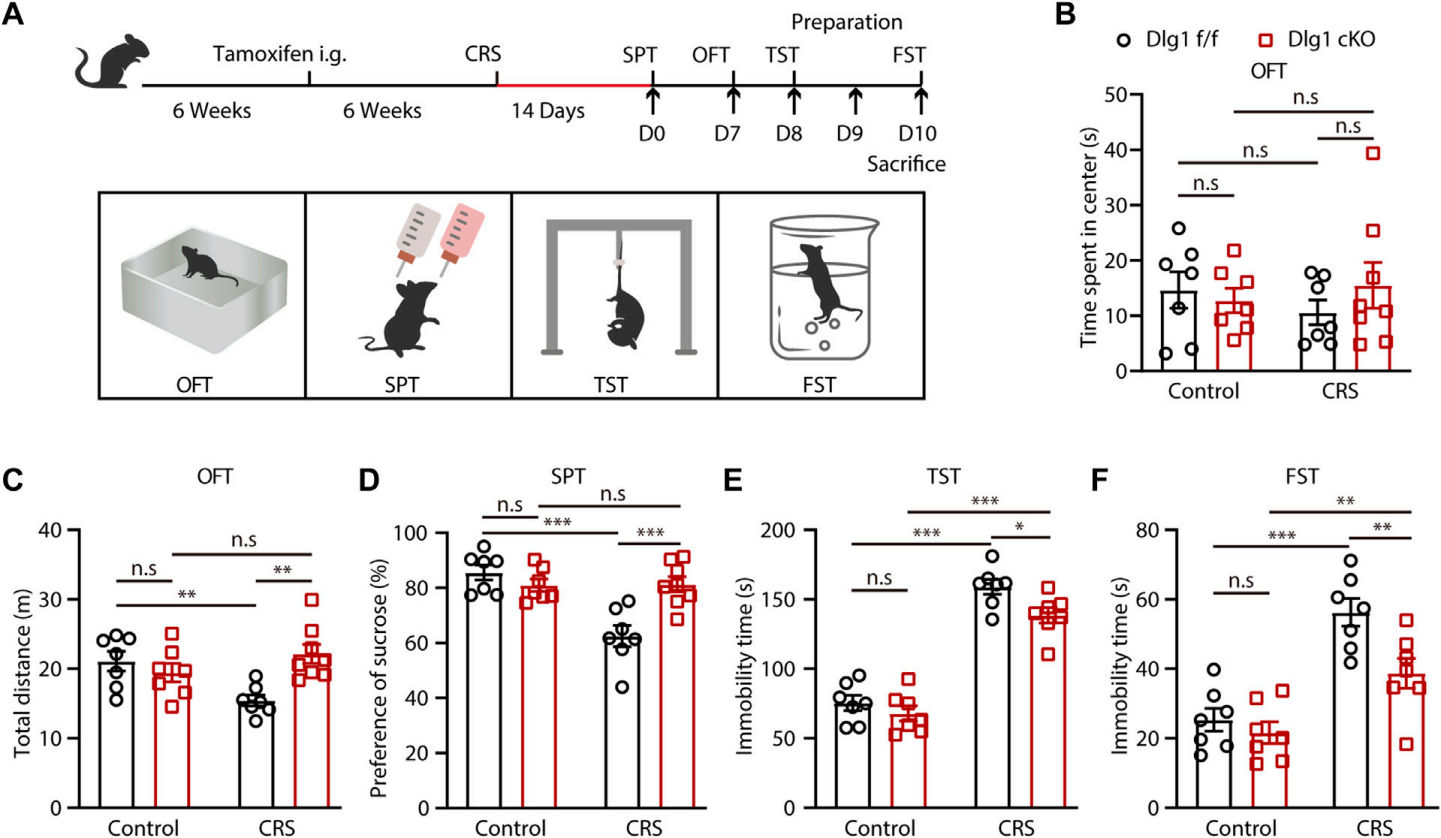
Microglial Dlg1 knockout alleviates CRS-induced depression-like behavior in mice. **(A)** The scheme of tamoxifen administration, stress stimuli and behavioral tests. **(B–C)** Travel-time spent in the central area and total travel-distance in the OFT of *Dlg1* f/f and *Dlg1* cKO mice with and without CRS exposure. **(D)** Sucrose preference of *Dlg1* f/f and *Dlg1* cKO mice with and without CRS exposure. **(E–F)** Immobility time of *Dlg1* f/f and *Dlg1* cKO mice with and without CRS exposure in the TST and FST. Data are presented as the mean ± SEM; *p < 0.05; **p < 0.01, ***p < 0.001, two-way ANOVA with Turkey’s multiple comparisons test.

To evaluate the role of microglial Dlg1 on depression, we established chronic restraint stress (CRS) model, which has higher etiological similarities to depression. Specifically, *Dlg1* cKO mice (*Dlg1* ^f/f^; CX3CR1creER) and their control littermates (*Dlg1* ^f/f^, term as *Dlg1* f/f) were subjected to tamoxifen at 6 weeks of age to induce microglia-specific knockout of *Dlg1*. These mice were then randomly assigned to either the control or CRS group. 6 weeks after tamoxifen administration, mice in the CRS group underwent chronic restraint, whereas mice in the control group were handled in a separate home cage at an equivalent length of time. After 2 weeks, behavioral tests, namely sucrose preference test (SPT), open field test (OFT), tail suspension test (TST), and forced swimming test (FST) were carried out (Figure 1A). We found that CRS mice exhibited no overt anxiety-like behavior (Figure 1B), but they displayed more prominent depression-like behaviors than non-stressed controls (Figures 1C–F). Stressed *Dlg1* f/f mice showed reduced locomotion in OFT (Figure 1C), less sucrose consumption in SPT (Figure 1D), and more immobility time in TST and FST (Figures 1E, F), indicating that the CRS model was established successfully. However, these depression-like behaviors induced by CRS were significantly alleviated in microglial *Dlg1* deleted mice. These results suggest that ablation of *Dlg1* in microglia ameliorates CRS-induced depression-like behavior.

### *Dlg1* ablation or CRS exposure has little effect on microglial density

**FIGURE 2:**
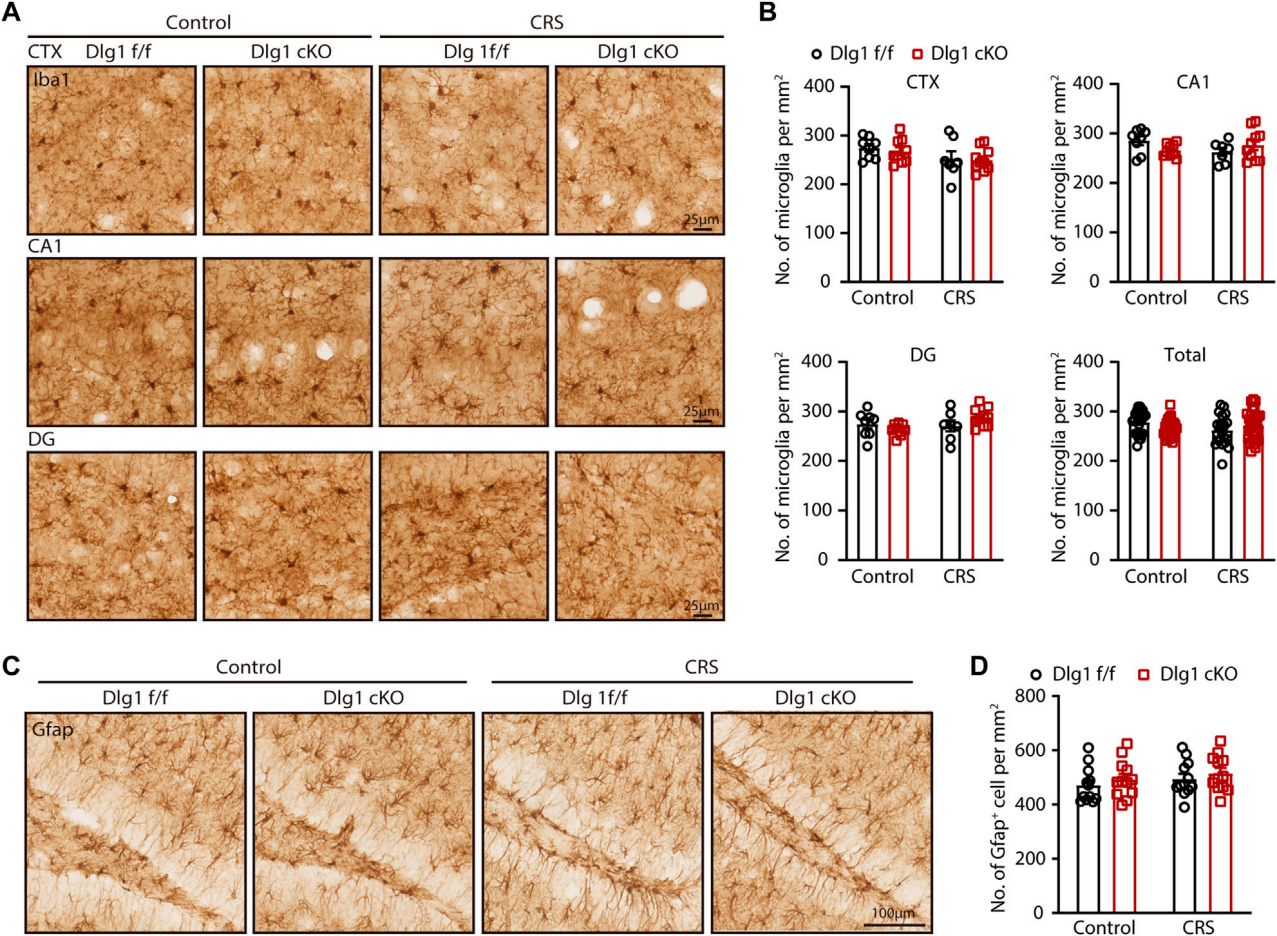
Microglial *Dlg1* knockout or CRS exposure has little effect on microglial density. **(A)** Immunohistochemical staining for Iba1 (microglia) of *Dlg1* f/f and cKO mice brain slices with and without CRS exposure in indicated brain regions. (Scale bars, 25 μm) **(B)** Quantifications of microglia density in indicated brain regions. **(C)** Immunohistochemical staining of Gfap (astrocytes) of *Dlg1* f/f and cKO mice brain slices with and without CRS exposure in DG (hippocampus). (Scale bars, 100 μm) **(D)** Quantification of astrocyte density in DG region. Data are presented as the mean ± SEM; *p < 0.05, **p < 0.01, ***p < 0.001, two-way ANOVA with Turkey’s multiple comparisons test.

After characterizing that specific knockout of *Dlg1* in microglia alleviates depression-like behavior, we next sought to investigate the effects of deleting *Dlg1* on microglia. To address this, we performed immunohistochemical staining of brain slices from *Dlg1* cKO and their control littermates in both control and CRS group (Figure 2A). The number of microglia in different brain regions, including the cortex (CTX), dentate gyrus (DG), and CA1 of the hippocampus, was nearly unchanged in *Dlg1* f/f mice subjected to CRS compared to *Dlg1* f/f mice in the control group. Deletion of *Dlg1* also showed no effect on microglia density in either the control or CRS groups (Figure 2B). Considering that astrocytes are known to communicate with microglia in a variety of disease states, in addition, we performed immunostaining for Gfap, a commonly used marker of astrocytes, and found that the number of astrocytes were not affected by CRS or deletion of *Dlg1* from microglia (Figures 2C, D).

### *Dlg1* deletion reverses microglial activated state found in mice with CRS exposure

**FIGURE 3:**
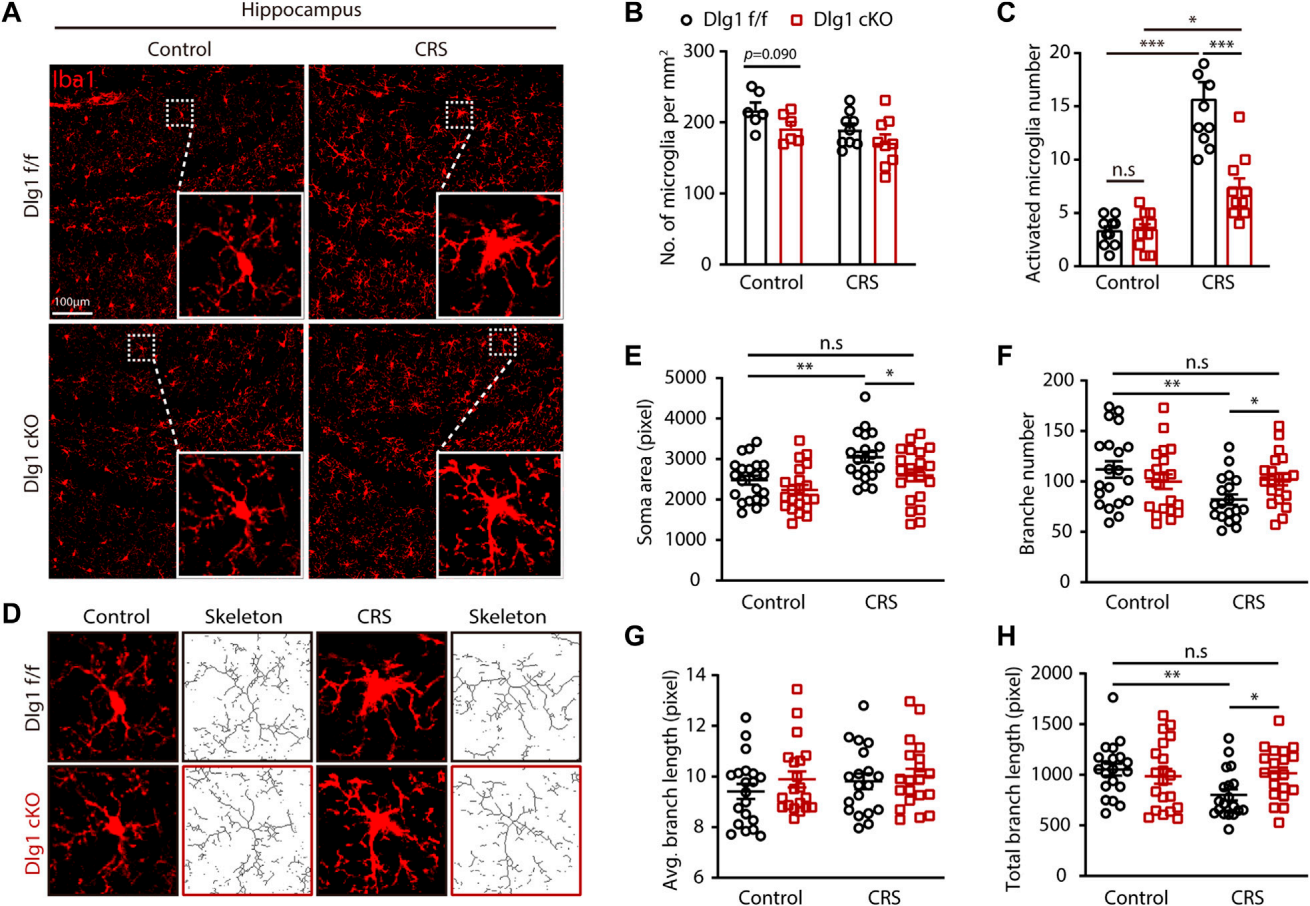
Microglial Dlg1 deletion reverses microglia overactive state found in chronic restraint stressed mice. **(A)** Immunofluorescence staining for Iba1 of *Dlg1* f/f and cKO mice brain slices with and without CRS exposure. **(B)** Quantification of microglia density in indicated four groups. **(C)** Quantification of the number of activated microglia in a single field of view. **(D)** Representative images of microglia (Iba1 immunostaining, red) and skeletonized microglia of *Dlg1* f/f and cKO mice brain slices with and without CRS exposure. **(E,F)** Quantitative analysis of microglial soma area **(E)**, branch number **(F)**, average branch length **(G)**, and total branch length **(H)**. Data are presented as the mean ± SEM; *p < 0.05, **p < 0.01, ***p < 0.001, two-way ANOVA with Turkey’s multiple comparisons test.

All four groups had compatible microglial densities, but some microglia in the CRS group exhibited alterations in morphology. In light of this, we performed immunofluorescence staining for Iba1, a microglia-specific marker, and found that the number of activated microglia, which possessed a larger volume of soma as well as shorter and bolder processes, were markedly increased in the *Dlg1* f/f mice subjected to CRS (Figures 3A–C). It is interesting to note that this increase was significantly reversed by microglia-specific knockout of *Dlg1* (Figure 3C). In light of the fact that microglia morphology and function are closely linked, we further analyzed the detail changes in microglia morphology (Figure 3D). We found that the soma area of microglia was notably increased while branch number and total branch length were markedly reduced in stressed *Dlg1* f/f mice, As expected, these activated morphology changes were significantly absent in *Dlg1* deleted microglia (Figures 3E–H). These results demonstrate that CRS provokes microglial activation, and knockout of *Dlg1* prominently reverses this effect.

### Stress-induced microglial inflammation was diminished by *Dlg1* deletion

**FIGURE 4:**
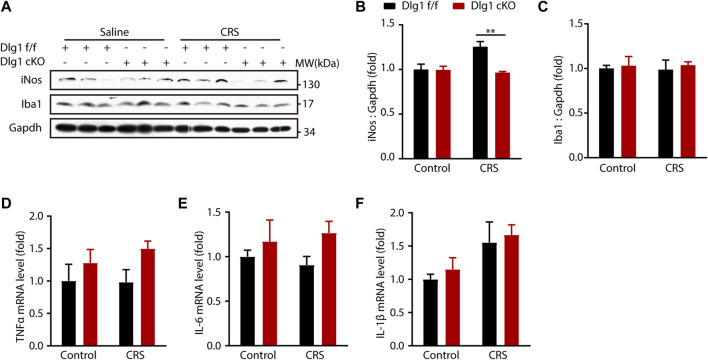
Microglial Dlg1 ablation partially decreases inflammation state in the mouse brain. **(A)** Immunoblots of iNOS, Iba1, and Gapdh in the hippocampus tissue of *Dlg1* f/f and cKO mice brain slices with and without CRS exposure. **(B,C)** Quantitative analysis of iNOS and Iba1 protein levels normalized to Gapdh. **(D–F)** RT-PCR analysis of *TNF-α*, *IL-6*, and *IL-1β* mRNA levels in the hippocampus tissues of *Dlg1* f/f and cKO mice brain slices with and without CRS exposure. Data are presented as the mean ± SEM; *p < 0.05, **p < 0.01, ***p < 0.001, two-way ANOVA with Turkey’s multiple comparisons test.

We next determined microglial status by examining the inflammatory cytokines. The protein level of inducible nitric oxide synthase (iNOS), termed as a marker of inflammation, was significantly upregulated in *Dlg1* f/f mice of CRS group, and *Dlg1* knockout reversed this upregulation (Figures 4A, B). In contrast, other cytokines like tumor necrosis factor *α* (TNFα), interleukin 6 (IL-6) and interleukin 1β (IL-1β) were found to have similar levels among four groups (Figures 3D–F). These results indicate that microglia display a mild activated state in CRS model, and deletion of *Dlg1* in microglia recruits its normal state.

## Discussion

In this study, we demonstrated that *Dlg1* ablation in microglia remarkedly reversed microglial activation and depression-like behavior in mice induced by exposure to CRS, indicating a potential target to suppress microglial activation, limit neuroinflammation, and thereby ameliorate depression.

Clinical researches demonstrate that perturbation of microglia homeostasis, like bacterial and viral infections [e.g., coronavirus (Mazza et al., 2020; Rogers et al., 2020), Ebola (Karafillakis et al., 2016)], and inflammation conditions in disease, such as stroke (Medeiros et al., 2020), neurodegenerative diseases (Galts et al., 2019), could contribute to major depression. These immune challenges induce microglial activation and cytokine production. Moreover, the cytokine levels in plasma are highly correlated with the severity of major depression and antidepressant responses (Miller et al., 2009; Black and Miller, 2015; Yirmiya et al., 2015). However, not all patients with depression display an overt inflammation. There are pathophysiological differences among each subtype of major depression (Lamers et al., 2013). In animal model of depression, an often-used immune challenge to establish depression model is lipopolysaccharide (LPS) (O'Connor et al., 2009), which provokes robust microglial activation and cytokine secretion and induces depression-like symptoms. Nevertheless, LPS administration does not cause prolonged anhedonia, lethargy or despair (DellaGioia and Hannestad, 2010). Given the pathophysiological features of different subtypes of depression, stress-induced depression model is needed to study the role of immune dysregulation in depression.

Our previous study adopted the LPS-induced model of depression, and we observed that microglia are activated, with a larger soma volume and shorter, bolder processes, and that microglial density is markedly increased (Peng et al., 2021). In contrast, in the current study, CRS-induced depression model, microglia activated in a much milder manner, without alterations in density, and morphological changes occur in only a fraction of microglia. Furthermore, cytokine levels in the brain that were significantly elevated in mice exposed to LPS showed comparable levels in mice with and without CRS exposure. Interestingly, however, deletion of *Dlg1* in microglia inhibits microglial activation and ameliorates depression-like behavior in both the LPS- and CRS-induced models of depression, prompting us to hypothesize that *Dlg1* deletion protects the animal from depression more than by targeting NF-κB signaling but restores microglia to homeostasis from a state of stress-induced dysregulation. Numerous studies have revealed that voltage-gated potassium channels are necessary for the activation of microglia, and Kv1.3 is particularly crucial among them (Pike et al., 2022; Zhang et al., 2022; Yuan et al., 2023). Kv1.3 potassium channel regulates microglial activation and is highly expressed in neuroinflammatory diseases (Rangaraju et al., 2017; Pike et al., 2022; Zhang et al., 2022; Yuan et al., 2023). Interestingly, Dlg1 interacts with the Kv1.3 through the PDZ binding domain (Tejedor et al., 1997). Moreover, Kv1.3 expression is downregulated upon *Dlg1* deletion in dendritic cells (Dong et al., 2019). These studies provide an insight into the potential mechanism by which Dlg1 regulates microglial activation. *Dlg1* knockout might maintain the microglial resting state by downregulating Kv1.3, which requires further study.

Emerging evidence demonstrates that exposed to chronic stress induces alterations in microglial density (Tynan et al., 2010; de Pablos et al., 2014; Kreisel et al., 2014). According to Tynan et al.’s study, the number of microglia was significantly elevated after CRS exposure. In our study, however, we found almost no alterations in microglia density between stressed and non-stressed Dlg1 f/f mice. Interestingly, according to previous studies, the effects of chronic stress on microglial activation are heterogeneous (Yirmiya et al., 2015). In addition to the proliferation of microglia, which is peaked at 2–4 days after stress, chronic stress also induces the apoptosis of microglia (Kreisel et al., 2014; Tong et al., 2017; Schramm and Waisman, 2022). After exposure to stress for several weeks, the microglia density even decreases in some specific brain region (e.g., DG of hippocampus) (Tong et al., 2017; Schramm and Waisman, 2022). In the present study, we sacrificed the mice after performing behavioral tests, and that was 10 days after mice were stressed, while animals were euthanized 24 h after the final stress session in Tynan et al. (2010)’s study. It is possible that the different time points had different effect on the numbers of microglia that were undergoing apoptosis, leading to the different results in microglial density. Additionally, the differences in the animal species, the exact brain regions, the CRS protocol, and the method that the number of microglia was determined may also account for the different outcomes.

Microglia are especially sensitive to the local environment. They react rapidly to what they encountered, thus making them not only a therapeutic target, but also a suitable indicator for monitoring the disease progression and therapeutic effect. Studies aiming at microglia inhibition have achieved promising results in alleviating depression. By blocking microglial activation with minocycline, depression-like behavior can be attenuated (Henry et al., 2008; Wang et al., 2020; Bassett et al., 2021). Clinical study provides more direct evidence in patients with unipolar psychotic depression by using minocycline as an adjunctive therapy (Miyaoka et al., 2012). Other anti-inflammatory drugs, like non-steroidal anti-inflammatory drugs (NSAIDs) also exhibit anti-depressive properties (Lehrer and Rheinstein, 2019). Our studies demonstrate that deletion of Dlg1 attenuates depression-like behavior in both LPS- and CRS- induced mouse models, providing a novel target for the development of treatment strategies.

## Data Availability

The original contributions presented in the study are included in the article/Supplementary Material, further inquiries can be directed to the corresponding authors.
